# The Treg/Th17 Paradigm in Lung Cancer

**DOI:** 10.1155/2014/730380

**Published:** 2014-04-29

**Authors:** Min-Chao Duan, Xiao-Ning Zhong, Guang-Nan Liu, Jin-Ru Wei

**Affiliations:** ^1^Department of Respiratory Medicine, The Eighth People's Hospital of Nanning, Nanning, Guangxi 530001, China; ^2^Department of Respiratory Medicine, The First Affiliated Hospital of Guangxi Medical University, Nanning, Guangxi 530021, China; ^3^Department of Respiratory Medicine, The First People's Hospital of Nanning, Nanning, Guangxi 530022, China

## Abstract

Pathogenic mechanisms underlying the development of lung cancer are very complex and not yet entirely clarified. T lymphocytes and their immune-regulatory cytokines play a pivotal role in controlling tumor growth and metastasis. Following activation by unique cytokines, CD4+ T helper cells differentiate into Th1, Th2, Th17, and regulatory T cells (Tregs). Traditionally, research in lung cancer immunity has focused almost exclusively on Th1/Th2 cell balance. Recently, Th17 cells and Tregs represent an intriguing issue to be addressed in lung cancer pathogenesis. Tregs play an important role in the preservation of self-tolerance and modulation of overall immune responses against tumor cells. Th17 cells directly or via other proinflammatory cytokines modulate antitumor immune responses. Notably, there is a close relation between Tregs and Th17 cells. However, the possible interaction between these subsets in lung cancer remains to be elucidated. In this setting, targeting Treg/Th17 balance for therapeutic purposes may represent a useful tool for lung cancer treatment in the future. The purpose of this review is to discuss recent findings of the role of these novel populations in lung cancer immunity and to highlight the pleiotropic effects of these subsets on the development and regulation of lung cancer.

## 1. Introduction

Lung cancer is the second most frequent cancer worldwide and continues to be the leading cause of cancer deaths [1]. Lung cancer is occurring in high frequencies in many economically developing countries; in the west the incidence is now declining which reflects changing cigarette smoking habits in the second half of the 20th century [1]. Only 15% of the patients survive for more than 5 years after primary diagnosis [1, 2]. Cigarette smoking and other noxious particles and gases that favor chronic lung inflammation have been established as risk factors for lung cancer development [2–4]. In particular, cigarette smoking with chronic inflammatory infiltrates in lung parenchyma [5], cigarette smoking with chronic obstructive pulmonary disease [6], and pulmonary tuberculosis [7] have been described as critical risk factors of lung cancer. In addition, tumor microenvironment consisting of immune cells is also identified as an indispensable participant of tumor immune pathogenesis [8].

Histologically, lung cancer is divided into two types: small cell lung cancer (SCLC) and non-small cell lung cancer (NSCLC). NSCLC represents about 80% of all lung cancer cases and includes three histological subtypes: squamous cell carcinoma, adenocarcinoma, and large cell carcinoma. About 80–90% of NSCLCs are directly related to tobacco smoke [9] while SCLC represents about 20% of lung cancers and nearly all SCLCs are associated with smoking [9, 10]. Several studies have demonstrated that tumor microenvironment consisting of immune cells is an indispensable participant of the neoplastic process by favoring tumor cell proliferation, survival, and metastasis [11, 12]. Lung cancer is more and more common and receiving increasing attention; however, lack of methods for early diagnosis and lack of systemic therapies are the main reasons why the prognosis for many patients is still poor. There is a need, therefore, to elucidate the immune mechanisms to develop new therapeutic strategies such as immunotherapy. However, the precise regulatory mechanisms of the disease are poorly understood.

Various studies have demonstrated that tumor-infiltrating lymphocytes, especially CD4+ helper T cells, are present in the lungs of patients with non-small cell lung cancer [13]. CD4+ T helper cells are significantly important in removing cancerous tissue or cells. CD4+ helper T cells can be functionally divided into Th1, Th2, Th17, and regulatory T cells (Tregs) based on the secretion of cytokines [14, 15]. They perform different biological functions in antitumor immunity and tumor immune evasion and play an important role, respectively, in tumor tolerance mechanisms, tumor immune microenvironment, and immune homeostasis [16, 17]. Traditionally, research in lung cancer immunity has focused almost exclusively on Th1/Th2 cell balance [18]. Recently, the identification of Th17 cells and Tregs not only changes the classical Th1/Th2 paradigm of T helper cell differentiation but also markedly facilitates our understanding of human immunity under both physiological and pathological conditions [15, 19, 20]. Notably, there is a close relation between Tregs and Th17 cells. With time, the relationship has become increasingly complex and more closely intertwined. Several studies showed that Th17 and Tregs are present in lung cancer [21, 22]; however, the possible interaction between these subsets in lung cancer remains to be elucidated. The aim of this review is to discuss recent findings of the role of these novel populations in lung cancer immunity and to highlight the pleiotropic effects of these subsets on the development and regulation of lung cancer. Targeting Treg/Th17 balance for therapeutic purposes may represent a useful tool for lung cancer treatment in the future.

## 2. Th1-Th2 Paradigm in Lung Cancer

Traditionally, naïve CD4+ T cells become activated and differentiate into two effector T cell subsets after encountering a specific antigen. Th1 cells, which produce interleukin (IL)-2, interferon-gamma (IFN-*γ*), and tumor necrosis factor-alpha (TNF-*α*), are the major effectors of phagocyte-mediated host defense, protective against intracellular pathogens, and Th2 cells, which produce IL-4, IL-5, IL-6, IL-13, and IL-10, are important in allergic responses and protection against infection of helminthic parasites [23, 24]. The key transcription factors driving Th1 cell differentiation are T-bet, STAT-1, and STAT-4, whereas the transcription factors STAT-6, c-maf, GATA-3, and NFAT are master regulators of Th2 development and function [14, 25]. IL-12 has been shown to induce the differentiation of Th1 cells and to enhance autoimmune disease in certain animal models [26]. Th1 cells secrete IL-2 and IFN-*γ* that suppress Th2 responses, whereas Th2 cells secrete IL-4 and IL-10 that inhibit Th1 responses [23]. Concerning Th1/Th2 paradigm in lung cancer, the hypothesis of Th1 predominance and downregulation of the Th2 response was reinforced by evidence from both murine studies and the clinical course of Th1 and Th2 based conditions in lung cancer. A study by Ito et al. [18] addressed the expression of Th1 and Th2 cells in lung cancer. It was found that the percentage of Th1 cells in tumor-infiltrating lymphocytes was significantly higher than in the corresponding peripheral blood and the proportion of Th2 cells was significantly lower than that of Th1 cells. This indicates that Th1 cells are dominantly tumor infiltrators. In addition, some studies showed that the mRNA and protein expression levels of INF-*γ* and IL-12 were significantly increased, whereas the expression level of IL-4 and the frequencies of the IL-4 variant −590T/C were decreased in lung tumor tissue [27–30]. All these studies demonstrated that these Th1 cells develop in the presence of tumor antigens and Th1 polarizing cytokines such as IL-12 and INF-*γ* in the lung microenvironment. Additionally, the Th1-to-Th2 ratios were significantly elevated in the tumor-infiltrating lymphocytes (TIL) of the patients with early stage lung cancer, while the Th1-to-Th2 ratios were significantly depressed in the PBL of the patients with tumor recurrences [18]. Moreover, the mRNA expression of IL-4 and IL-10 in tumor tissue and pleural effusion of NSCLC patients was significantly higher than that of IL-2, IL-12, and INF-*γ* [31]. These findings suggested that the imbalance and conversion of Th1 and Th2 cells might be responsible for both the occurrence and the progression of lung cancer.

Although the above findings have suggested that Th1 and Th2 cells are involved in lung cancer development, the functional role of Th1/Th2 tumor-infiltrating T cells is not clear. Experiments performed on tumor bearing mice showed that T-bet knockout mice have significantly higher tumor load associated with reduced Th1 cells, suggesting that T-bet expressing Th1 cells protect from lung tumor growth [32]. IL-10 transgenic mice are unable to limit the growth of immunogenic tumors; however, administration of blocking IL-10 mAbs restored in vivo antitumor responses [33]. IFN-*γ* may exert potent antitumor effects on lung cancer and metastasis, as this cytokine boosts natural killer cell activity, induces macrophage activation and antigen presentation, and activates tumor-specific CD8+CTLs which are required for the elimination of cancer cells [34–36]. IL-10 inhibits the generation of cell-mediated antitumor immunity by inhibiting a broad array of immune parameters including antigen presentation, antigen-specific T cell proliferation, and type 1 cytokine production [37]. Together, these findings support the implication of enhanced Th1 cells in augmenting antitumor responses but enhanced Th2 cells in downregulating antitumor immunity.

Of note, although the Th1 and Th2 responses can be seen as discrete responses in lung cancer development, there is considerable cross-talk and overlap between the functions of the two subsets. Previous studies have shown that peripheral blood lymphocytes from NSCLC patients with recurrence showed an unfavorable imbalance between Th1 and Th2 cells, with significantly depressed Th1-to-Th2 ratios [18]. Similarly, tumor cells from patients with advanced lung cancer express some type 2 cytokines such as IL-10 and transforming growth factor-*β* (TGF-*β*), while little or no levels of type 1 cytokines such as IL-2 and IFN-gamma were noted [38, 39]. With the progression of a tumor, including malignant effusion and distant metastasis, the cell-mediated immunity of lung cancer patients is impaired and tumor cells produce type 2 cytokines to suppress the differentiation of T cells into Th1 cells. In addition, these immunosuppressive cytokines IL-10 and TGF-*β* might not only suppress Th1 cell responses by tumor-infiltrating T cells but also favor the development of regulatory T cells [40–42].

## 3. Treg Cells in Lung Cancer

Th17 and regulatory T cells (Tregs) have replaced the 20-year-old Th1-Th2 paradigm [43]. This new paradigm has significantly improved our understanding on the differentiation of functional CD4+ T helper cell subsets and T cell regulation of inflammation and autoimmunity. Studies ongoing for more than a decade have provided firm evidence for the existence of a unique CD4+CD25+ T-cell population of “professional” regulatory/suppressor T cells that actively and dominantly prevent both the activation and the effector function of autoreactive T cells that have escaped other mechanisms of tolerance [44–46]. TGF-*β*1 and IL-2 are the two crucial cytokines involved in the differentiation of naïve T-cells into Tregs, which express the forkhead lineage-specific transcription factor FoxP3 protein. TGF-*β*1 together with IL-2 is also needed for Tregs expansion. IL-2-induced STAT5 has a prominent role in promoting Foxp3 expression [47]. All-trans retinoic acid (atRA), an active metabolite of retinoic acid, markedly enhances TGF-*β*-induced Foxp3 expression and stability in mice [48] and the expansion of these Tregs [49]. IL-2-deficient mice have a reduced number of nTreg-cells, indicating the importance of Treg cell proliferation [50]. Multiple lines of evidence have indicated that Tregs are involved in the control of the local immune response and in the growth of human lung cancer [21, 51, 52]. A higher level of TGF-*β*1 in the BALF of patients with primary lung cancer compared with the healthy subjects has been found [53]. In addition, IL-2 levels are higher in patients with NSCLC [54] compared with healthy controls. The findings of higher levels of both TGF-*β*1 and IL-2 suggest that these proinflammatory cytokines might promote the generation and differentiation of Tregs in lung cancer. Furthermore, it was found that there were increased proportions of tumor-infiltrating CD4+CD25+ T cells in patients with early stage NSCLC, and these CD4+CD25+ T cells were found to secrete immunosuppressive cytokine TGF-*β*, which may play a role in cancer progression [55, 56]. In addition, these regulatory T cells have been shown to express cytotoxic lymphocyte-associated antigen-4 CTLA-4 (CD152) in mice [57], and triggering of CTLA-4 has been shown to induce TGF-*β* secretion [58]. Similarly, Chen et al. [59] also found that increased proportions of CD4+CD25+ T cells in malignant pleural effusion (MPE) were regulatory T cells as they express high levels of Foxp3 transcription factor and CTLA-4. Moreover, pleural CD4+CD25+ T cells could potently suppress the proliferation of CD4+CD25- T cells and CTLA-4 was involved in the suppressive activity of pleural CD4+CD25+ T cells. In a recent study, Ganesan et al. [60] demonstrated that tumor-infiltrating Tregs partially repressed CD8+ T cell responses in mouse models of lung adenocarcinoma. Thus, Treg cells in NSCLC appear to selectively inhibit host immune responses and therefore might contribute to cancer progression. What underlies the enrichment of Treg cells within tumor tissue or PE of lung cancer patients? There is considerable evidence to suggest that increased CD4+CD25+ T cells in tumor site might be due to either active recruitment or local differentiation. A study by Zhao et al. [61] showed that there were increased levels of TGF-*β*1 and IL-2 in serum in patients with NSCLC compared with healthy controls. TGF-*β* induces Treg expansion in lung cancer microenvironment [48]. In addition, other studies identified that CCL22 in MPE might be related to the accumulation of Treg cells in MPE. Indeed, an in vitro migration assay further confirmed that MPE could induce the migration of Treg cells and that either anti-CCL22 mAb significantly inhibited the ability of the MPE to stimulate Tregs chemotaxis [62]. Further analysis of Tregs and related cytokines in lung cancer patients and tumor bearing animals clearly demonstrated the relationship between the stage of the disease and the relative proportion and number of Treg cells. For instance, experimental results suggested that Tregs were shown to inhibit NK cell-mediated suppression of tumor growth and metastases largely by a TGF-*β*-dependent mechanism [63]. Petersen et al. [64] reported that patients with stage I NSCLC who have a higher proportion of tumor Treg cells had a significant risk of recurrence. Similarly, Shimizu et al. [65] also showed that tumor-infiltrating FoxP3+ Tregs correlate with cyclooxygenase-2 (COX-2) expression and increased tumor recurrence in stage I to stage III NSCLC. Furthermore, Liu et al. [66] showed that a high FoxP3+ Treg/CD8+ T cell ratio is a risk factor for poor response to platinum-based chemotherapy in advanced NSCLC. This means that Tregs are able to inhibit antitumor immunity and mediate immune tolerance favoring tumor growth. However, to the best of our knowledge, although the elevated frequency of Tregs in tumor tissues correlated with the prognosis, whether or not Tregs directly contribute to tumor growth remains unclear. Further research should be done by using adoptive transfer of Treg cells and IL-2 or TGF-*β*-null (or IL-2 or TGF-*β* deficient) mice.

## 4. Th17 Cells in Lung Cancer

The subset of CD4+ T cells that produce both IL-17A and IL-17F is now defined as a separate subset of Th17 cells. Distinct from Th1 and Th2 cells, Th17 cells are reported to be generated from naïve T cells by IL-6, IL-1, and IL-21, with or without TGF-*β*, and are expanded and stabilized further by IL-23 [67–70] and by virtue of expressing the orphan nuclear receptors RORgt and ROR *α* as critical transcription factors [71]. STAT3 regulates IL-6-induced expression of RORgt and RORa and IL-17 production [72]. In the mouse, naïve CD4+ T cells stimulated by TGF-*β* and IL-6 differentiate into Th17 cells [73]. However, whether TGF-*β* plays a decisive role in the differentiation of naïve human CD4+ T cells into Th17 cells is controversy. Acosta-Rodriguez et al. [74] found that human Th17 cells originate in response to the combined activity of IL-1*β* and IL-6, whereas Wilson et al. [75] found that the activity of IL-1*β* or IL-23 alone was critical. In some human studies, the addition of TGF-*β* to human naïve or memory CD4+ T cells was even found to be inhibitory on the development of Th17 cells [76]. Many studies have demonstrated the importance of Th17 cells in clearing pathogens during host defense reactions and in inducing tissue inflammation in autoimmune disease; however, the role of Th17 cells and related cytokine IL-17 in lung cancer immunity has been confusing.

Besides IL-17 and IL-17F, Th17 cells also secrete other cytokines such as IL-21, IL-22, and CCL20 [76]. IL-17 (also known as IL-17A) was first cloned in 1993 and identified as cytotoxic T lymphocyte-associated antigen (CTLA)-8 [77]. IL-17F was later discovered and closely related to IL-17A [78]. They are mainly produced by activated memory CD4+ T cells but can be induced in CD8+ T cells, NK T cells, DC cells, and possibly other cells [79–84]. An induction of IL-17A mRNA and protein expression was noted in lung CD4+ T cells in patients with NSCLC as compared to healthy controls, suggesting IL-17 is involved in lung cancer [22, 31]. However, the role of IL-17 in tumor immunity remains undefined. Functionally, overexpression of IL-17 in tumor cell lines promotes angiogenesis and tumor growth when the tumors are implanted in immune-compromised mice [85]. Intranasal treatment of mice with a neutralizing anti-IL-17A antibody in experimental lung adenocarcinoma caused a significant reduction of tumor growth as compared to control treated mice [32]. Similarly, IL-17A deficiency or IL-17A blockade led to suppression of lung metastasis in tumor models [86]. Further, IL-17 could directly promote the invasion of NSCLC cells both in vitro and in vivo. Furthermore, the elevated expression of IL-17 in peripheral blood was associated with the TNM stage [87]. These reports indicate that IL-17-mediated responses promote tumor development through the induction of tumor-promoting microenvironments at tumor sites and that IL-17-mediated regulation of myeloid-derived suppressor cells (MDSCs) is a primary mechanism for its tumor-promoting effects [88]. In contrast, recent reports indicate that tumor growth in subcutaneous tissue and lung tumor metastasis are enhanced in IL-17^−/−^ mice and that the mechanism is associated with reducing IFN-g-producing tumor-infiltrating NK and T cells [89, 90]. It implicates that IL-17-mediated responses are protective against tumor development. With regard to IL-22, several studies have showed that high expression concentrations of IL-22 were detected in tumor tissue, MPE, and serum of patients with lung cancer and that the overexpression of IL-22 was correlated with occurrence and progress of lung cancer [91, 92]. IL-22 might also play a role during tumor genesis because IL-22 stimulates signaling pathways that are involved in the cell proliferation, cell apoptosis, and cell cycle control [93–96]. So far, however, additional roles for the Th17-derived cytokines in lung cancer remain largely unexplored. Additionally, further research should be done by using adoptive transfer of Th17 cells in mice to demonstrate the cellular mechanisms.

Concerning Th17 cells in lung cancer, recent data from humans and mice clearly support the role of Th17 cells in lung cancer pathogenesis. It was found that the expression of Th17 markers ROR c and ROR *γ*t was significantly induced in NSCLC [31], and a larger Th17 cell was included in SCLC [97]. Consistently, Th17 numbers in malignant PE from patients with lung cancer were much higher than numbers in peripheral blood. The overrepresentation of Th17 cells in MPE might be due to Th17 cell differentiation and expansion stimulated by pleural proinflammatory cytokines IL-1*β*, IL-6, IL-23, or their various combinations and to recruitment of Th17 cells from peripheral blood induced by pleural chemokines CCL20 and CCL22 [98]. In spite of the above findings highlighting the expression and differentiation of Th17 cells in lung cancer, their physiological functions in cancer immunity still remain largely unknown. It has been suggested that Th17 cells themselves do not have direct in vitro killing activity to tumor cells. Th17 cells stimulate tumor residential cells to produce CCL2 and CCL20, which provokes the recruitment of dendritic cell, granulocyte, CD4+ T cell, CD8+ T cell, and NK cell to the tumor site [99]. Increased numbers of DCs after Th17 cell transfer enhance tumor antigen in the lung and migrate to the lymph nodes where they activate CD8+ T cells against the tumor [100]. The tumor-specific CD8+ T cells may kill tumors independent of IFN*γ*, possibly via perforin pathway [28]. Taken together, these reports demonstrate that Th17 cells participate in antitumor immunity by facilitating T cell recruitment to the tumor site and CD8+ T cell priming and suggest a new avenue for developing Th17 cell-based therapy for lung cancer.

## 5. The Balance and Correlation between Th17 Cells and Treg Cells in Lung Cancer

Besides the above-mentioned difficulty to clarify the effective role played by Th17 and Treg cells in lung cancer pathogenesis, recent studies made this matter even more complex providing the clue of these T-cell subsets [31, 101, 102]. Recently, it became increasingly clear that CD4+ T cell subsets are not stable and display plasticity during differentiation and maintenance [47]. There is a close relation between Tregs and Th17 cells. In mice these cells originate from a common precursor, with the differentiation of which is dependent upon the production of dendritic cells activated by microorganisms [103]. Moreover, the progenitor cells differentiate to Th17/Treg intermediate cells, which express both RORC and Foxp3 [104]. Additionally, Tregs and Th17 cells share common chemokine receptors (CCR6, CCR4) and homing properties (CCL20) [105]. In human a differentiation link between Th17 cells and Tregs has been reported, in which TGF-*β* is essential for the generation of both cells [103]. The differentiation of Th17 cells is inhibited by high TGF-*β* concentrations but requires IL-1*β* and IL-6. Retinoic acid, which is a key regulator of TGF-*β* dependent immune responses, is able to inhibit ROR-*γ*t in Th17-inducing conditions and simultaneously promote Tregs differentiation [103, 104]. Besides, there is also an inverse correlation between ROR-*γ*t and FoxP3 [106]. Indeed, in the presence of proinflammatory cytokines and low concentration of TGF-*β*, ROR-*γ*t expression is further upregulated, whereas FoxP3 expression and function are inhibited. This evidence shows the importance of cytokines environment in the differentiation of CD4+ T-cell subsets, depending upon the balance of expression of the transcriptional factors ROR-*γ*t and FoxP3. Recently, the relationship has taken a further twist, with the surprising finding that Tregs are able to convert to Th17 cells in the context of inflammatory signals, such as IL-1*β*, IL-6, IL-21, and IL-23 [107–109].

Many studies have revealed that lung cancer may occur as a consequence of cytokine imbalance and eventually of the Th17/Treg ratio. For instance, SCLC as well as NSCLC cells overexpress TGF-*β* [110, 111]. Serum levels of TGF-*β* were increased in lung cancer patients with lymph node metastasis compared with patients who were without lymph node metastasis, and the TGF-*β* levels were significantly higher in patients with stage III disease compared with patients who had stages I and II disease [112]. Activated TGF-*β* promotes tumor metastases [113]. Notably, TGF-*β* has suppressive activity in early tumorigenesis but may become tumor-promoting in the later stages of the disease [113]. Similarly, IL-17A may elicit pro- as well as antitumor properties. Additionally, this cytokine induces IL-6 production to interfere with Tregs development. Studies by Zhou et al. [108] have shown that there is a significant increase in Tregs and FoxP3 expression and a decrease in Th17 cells, ROR ct and IL-17 expression in peripheral blood of NSCLC patients while compared to that in healthy patients, and Foxp3 levels correlated with levels of RORc and IL-17. In particular Th17/Treg ratio is negatively correlated with the TNM stages. As a consequence, tumor-derived low TGF-*β* may synergize with IL-6 and IL-21 to promote Th17 cell differentiation in early stage lung cancer, while in late stage disease tumor-derived high TGF-*β* may induce overproduction of Treg cytokines and, in turn, promotes a shift in the Th17/Treg balance toward a Treg response and inhibiting the Th17 response [108, 114]. Further study by Ye et al. [101] provides functional evidence that regulatory T cells from malignant pleural effusion in lung cancer were found to inhibit generation and differentiation of Th17 cells via the latency-associated peptide LAP. Thus, Tregs and Th17 arise in a mutually exclusive fashion, depending on tumor microenviroment. Taken together, these results suggest that Tregs and Th17 cells are involved in the perpetuation of the inflammatory immune response in lung cancer, and restoring an adequate cytokine network and Th17/Treg balance may help to achieve a better clinical response.

## 6. Closing Remark and Prospective

T lymphocytes and related cytokines modulate immune responses in the tumor microenvironment during progression/metastasis, and the balance between destructive inflammation and protective immunity determines the direction of the malignant process [115–119]. Th17 and Treg cells are two mutually contradictory T cell subsets. The differentiation of Th17 cells depends on the concomitant action of IL-6 and the suppressive cytokine TGF-b which is also necessary for the induction of Tregs. IL-6, in turn, inhibits the development of Tregs suggesting that IL-6 plays a pivotal role in dictating the balance between the generation of Tregs and Th17 cells. Maintaining an appropriate balance between Th17 and Treg cells can ensure effective immunity while avoiding inflammatory and tumor immunosurveillance. Accumulated evidence has demonstrated quantitative or functional imbalance between Th17 and Tregs and these subsets' expression correlation with prognosis in lung cancer, suggesting that Th17 and Tregs represent important key pathogenic players in lung cancer pathogenesis. Th17 cells dominantly act to induce antitumor immunity. In contrast, Tregs better enable inhibition of antitumor immunity. However, the molecular mechanisms underlying the involvement and regulation of these two subsets in lung cancer immunopathology remain largely unknown. In addition, a number of crucial questions remain to be answered. What precise roles do TGF-*β*, IL-6, IL-17, and IL-22 play in lung cancer immunopathology? How might Th17/Treg imbalance be induced and lead to lung cancer immune pathogenesis? Is the Th17/Treg imbalance in early stages of tumor development the same as in late stage? Are there complementary roles for Th17 and Treg responses in lung cancer? Further understanding of the mechanisms of Th17/Treg-mediated inflammatory immune responses, in tilting the balance between destructive inflammation and antitumor immunosurveillance, may open new lines of investigation for lung cancer treatment in the future.
